# Preliminary study of donor volume changes after dual-graft liver transplantation in rats

**DOI:** 10.3389/fimmu.2023.1183426

**Published:** 2023-10-16

**Authors:** Dan Wang, Yanling Ma, Baohong Gu, Xuemei Li, Yang Yu, Ying Zhang, Hao Chen

**Affiliations:** ^1^ The Second Clinical Medical College of Lanzhou University, Lanzhou, China; ^2^ Department of Laboratory Medicine, The First Medical Centre, Chinese PLA General Hospital, Beijing, China; ^3^ The Department of Tumor Surgery, Second Hospital of Lanzhou University, Lanzhou, China; ^4^ The Key Laboratory of The Digestive System Tumors of Gansu Province, Second Hospital of Lanzhou University, Lanzhou, China

**Keywords:** liver transplantation, volume, dual graft, rat, atrophy

## Abstract

Dual-graft liver transplantation (DGLT) expands the pool of donors, ensures the safety of the donors, and treats a potential small for size syndrome (SFSS). However, some of the recipient graft showed atrophy. The cause and mechanism of the unbalanced proliferation and atrophy of dual grafts after clinical DGLT have not been clarified. We established and optimized the rat model of DGLT to explore the causes of growth unbalance. Continuously and dynamically observed bilateral graft volume and portal vein blood flow change by magnetic resonance imaging (MRI) and ultrasound (US). We detected liver function indexes: alanine aminotransferase (ALT), aspartate aminotransferase (AST), lactate dehydrogenase (LDH), total bilirubin (TBIL), direct bilirubin (DBIL), and indirect bilirubin (IBIL). Liver samples from receptors were obtained for morphology, and apoptosis was measured by RT-PCR and western blot. Optimization of the model improved the 7-day survival rate from former 58.3% to 87.5%, and the 30-day survival rate was 68.8%. The volume of the right graft gradually increased, and the left graft atrophied during the 30-day observation period. The portal blood flow of the left graft gradually decreased until the 30th day (0.13 ± 0.01 ml/s) compared with the sham group (0.63 ± 0.05 ml/s), and the right graft significantly increased on the 30th day (0.75 ± 0.11ml/s). The liver function initially increased and then recovered. The total volume (12.52 ± 1.60 ml vs 4.47 ± 0.08 ml) and weight (12.09 ± 1 g vs 4.91 ± 0.18 g) of the graft increased significantly compared to pre-transplantation and reached the level of the sham operation group on the 30th day. The volume and weight of the right graft increased more than those of the left graft (*P* < 0.05). There was more inflammatory cell infiltration in the left graft, and the right graft had obvious proliferation of hepatocytes and mature bile duct cells. Left grafts were more prone to apoptosis than right grafts (*P* < 0.05). In conclusion, growth of the right graft is superior to the left; after double liver transplantation, perfusion blood flow and apoptosis may be the reason contributing to the volume differences in dual grafts.

## Graphical Abstract

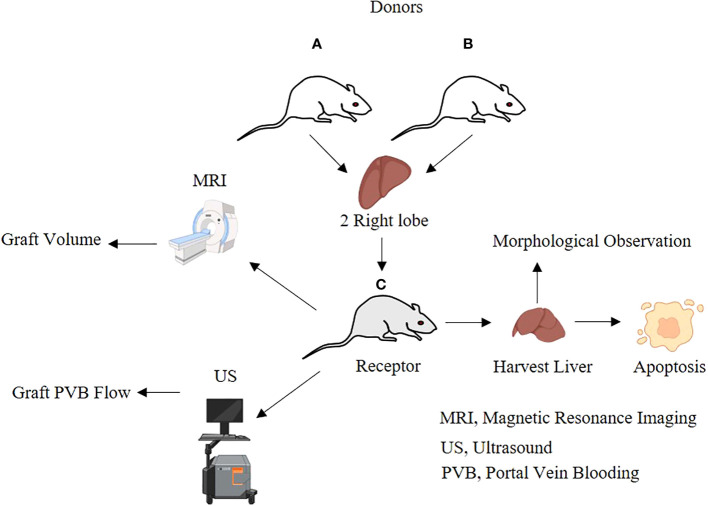

## Introduction

For many end-stage hepatobiliary diseases, liver transplantation is the most effective treatment strategy (1–3). However, the main obstacle with regard to liver transplantation is donor shortage (4–6). Viral hepatitis and end-stage liver disease are very prevalent worldwide (7, 8), but organ donation rates remain low. Organ shortages caused by imbalance in supply and demand are a common problem and a challenge (9). Subsequently, the emergence of living donor liver transplantation (LDLT) has been a great milestone and therefore greatly increased the number of donors within a considerable time frame (10). The main limitation of LDLT is the insufficient size of the graft (11, 12). Usually, left lobe graft from small donors cannot meet the metabolic needs of larger liver recipients (4, 13). To curb this problem, right lobe implantation is always the most appropriate and best for surgical purposes (4). However, donor safety in liver transplantation is often the most important precaution and consideration (14). Although right lobe donation can meet the needs of recipients, the remaining left lobe sometimes endangers the donor due to small size and insufficient residual liver function (13). In this case, the donor is not allowed to donate the right lobe (13, 15–18). In addition, the size of the donor and recipient liver grafts does not match, and the fatty degeneration of the donor and the complex anatomical structure of the right lobe restricts the acquisition of grafts (15).

Based on this problem, Lee achieved the first dual-graft liver transplantation (DGLT) in 2001 (13). Furthermore, DGLT in Europe, Japan, and China has made remarkable progress (**Table 1**). Compared to traditional liver transplantation, the striking feature is that it requires two donors, which makes the donor safer because it theoretically reduces the donor’s donated liver volume. Henceforth, it meets the receptor’s physiological functions of the liver and reduces the donor’s risk to hazardous conditions simultaneously (14). The main reason is that the graft provided by two donors could provide the recipient with a relatively sufficient liver, improve the survival rate of the recipient, and reduce complications (19). The mismatch of the blood type between a donor and a recipient did not have any prognostic effects on the patients’ condition (26). In addition, based on the dual-donor characteristics of DGLT, some experts have proposed that a donor bank can be established among social groups and that donors can be flexibly matched to achieve optimal therapeutic effects (25). However, DGLT also has many challenges and problems. For example, the surgical procedure is very complicated and can only be performed in advanced well-equipped medical centers, which alters the risk from a single donor to two donors (14). More importantly, in clinics, some DGLT has proven to have one-sided graft atrophy post-surgery in some patients (13, 20, 24, 27, 28). Reports have found that atrophied grafts mainly include the following factors: (1) The portal vein of the graft with a larger volume receives more blood flow and presents with a better proliferative state than the small-volume graft, which is called “blood stealing” (21, 23, 29). (2) DGLT includes four transplantation modes: left hepatic lobe to left hepatic fossa, right hepatic lobe to right hepatic fossa, left hepatic lobe to right hepatic fossa, and right hepatic lobe to left hepatic fossa; the combination of the two grafts depends on the occasion (13, 14). Studies have found that left hepatic lobe to right hepatic fossa and right hepatic lobe to left hepatic fossa are related to the atrophy of grafts; grafts not in the original hepatic fossa are prone to tension, distortion, stretching, and atrophy (16, 20). In addition, studies have shown that hepatic vein blockage is also related to liver graft atrophy (22, 30–32). If one side of the hepatic vein is blocked, blood flow does not proceed smoothly or the flow rate is slow compared with the other side of the graft hepatic vein (30). The blood vessel pressure from that side of the liver is always high and causes the portal vein velocity to decrease (30). Ultimately, the total portal vein blood flow to the contralateral portal vein increases, causing the contralateral graft to mature well (30, 33). Therefore, the main aim and purpose of our study are to explore the factors that affect the volume difference of dual grafts post-transplantation.

**Table 1:** Reported cases of dual liver transplantation worldwide.

Author	Region	Time	Reference	Cases
Song et al.	Korea	2017 (19)	Ann Surg	400
Broering et al.	German	2007 (20)	*Liver Transpl*	2
Kaihara et al.	Japan	2002 (21)	*Surgery*	1
Nicoluzzi et al.	Brazil	2012 (22)	*Rev Col Bras Cir*	1
Botea F, et al	Romania	2013 (23)	*Chirurgia (Bucur)*	1
Dayangac et al.	Turkey	2010 (16)	*Transplant Proc*	1
Zhang et al.	China	2008 (15)	*Hepatogastroenterology*	1
Chen et al.	China	2009 (24)	*J Surg Res*	6
Yang et al.	Taiwan	2009 (25)	*Surgery*	4

## Materials and methods

### Ethical approval of this study’s protocol

The present study was approved by the Laboratory Animal Protection and Use Committee of Lanzhou University of China (ID:2016-D42), and all experiments were conducted according to the government and international guidelines regarding animal experimentation. Animal suffering was minimized, and the total number of rats was limited so that few were utilized for easy control and experimentation and to avoid wastage.

### Animal and experimental design

All SD (Sprague Dawley) rats were provided by Lanzhou Veterinary Research Institute of the Chinese Academy of Agricultural Sciences under the production license number SYXK (Gan) 2015-0001 [laboratory animals use license number SYXK (Gan) 2018-0003]. All animals were housed under standard conditions with a 12-h light/dark cycle, with free movement permitted, a constant temperature and humidity, and ad libitum access to water and food. The rats were allowed to acclimatize to the new environment for ≥3 days, and each rat was fasted for 12 h before surgery. After the experiment, all rats were euthanized by cervical dislocation, and respiratory movements and heartbeats were monitored to verify that the rats had demised.

A total of 96 SPF male SD rats, 8–10 weeks old, weighing 270 g–300 g, were randomly divided into transplantation groups (transplantation group, n = 72) and sham groups (sham group, n = 24). The transplantation group (n = 72) randomly used two donors and one recipient for transplantation, and the dual right upper lobes (45%–50% of the recipient liver weight) were transplanted in one recipient. There were 12 recipients assigned to collect specimens and perform hemodynamic, morphological, and serological monitoring, while the other 12 recipients were observed until the 30th day after the operation for survival analysis. The sham group (n = 24) was used to mobilize the liver by the division of the hepatic ligaments. There were 12 rats in the sham group designated for specimen collection, and the remaining 12 rats were used for survival analysis.

Basic data of the dual-graft liver transplantation rat model are depicted in **Table 2**.

**Table 2:** Basic data of the dual-graft liver transplantation rat model.

Dual liver transplantation	Data
Donor weight	274.5 ± 3.4 g
Recipient weight	287.2 ± 5.7 g
GRLWR	54.0%–64.6%
Graft weight	5.98 ± 0.17 g
Recipient liver weight	10.14 ± 0.42 g
Operation time	2 h
Cold ischemia time	35 min
Warm ischemia time	15 min
Anhepatic phase	25 min

### Transplantation procedure

**Figure 1:**
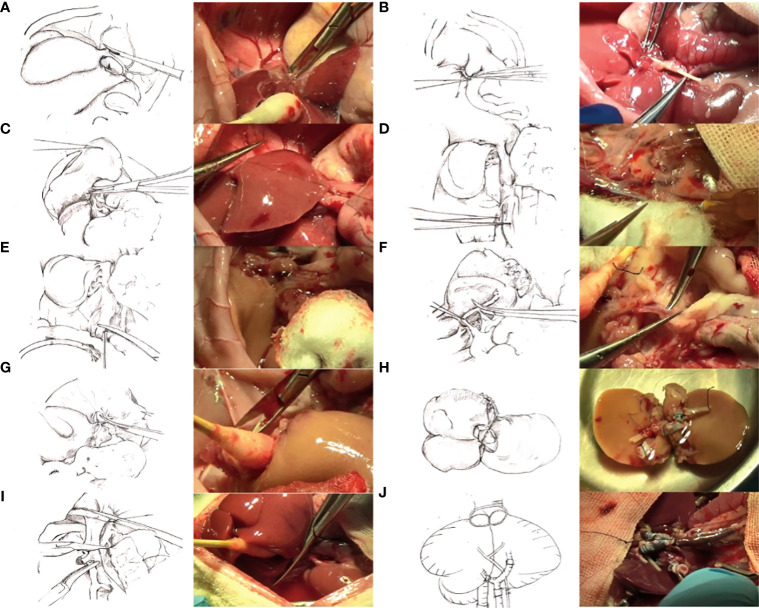
**(A)** Ligation of the left axillary vein. **(B)** Bile duct intubation. **(C)** Excision of access liver lobes and preservation of the right upper lobe. **(D)** Exposition of the inferior cavity and ligation of the right renal vein and right adrenal vein. **(E)** Systemic heparinization. Irrigation of the liver through the abdominal aorta changed the color to gray. **(F)** Exposure and cutting of the portal vein. **(G)** Cutting of the thoracic aorta. **(H)** Connection of two grafts. **(I)** Removal of recipient liver. **(J)** Transplantation of the composite donor into the recipient, opening of the portal vein, and changing of the graft into red.

The entire surgical process is shown in **Figure 1**, including harvesting of the donor’s liver, Y-shaped blood vessel acquisition, graft trimming, and dual-graft connection as well as surgery involving the recipient.

### Measurement of the liver volume by MRI

Dual- graft volume data in dual grafts on the 1st, 3rd, 7th, and 30th days post transplantation were acquired on a Siemens Verio 3.0 T scanner (Siemens, Erlangen, Germany).

### Determination of portal vein blood flow using an ultrasound imaging technique

Ultrasonic detection of portal vein velocity (PVV, portal vein diameter), portal vein diameter (PVD), and portal vein blood flow (PVF, portal vein) on the 1st, 3rd, 7th, and 30th days after transplantation was executed (Siemens Acuson Sequoia, probe: 10L4, frequency of the transducer: 4–10 MHz). Because the hepatic artery was ligated during the transplantation process, the hepatic artery hemodynamics could not be measured.

### Serum parameter

Preoperatively and 3, 7, and 30 days after operation, alanine aminotransferase (ALT), aspartate aminotransferase (AST), lactate dehydrogenase (LDH), total bilirubin (TBIL), direct bilirubin (DBIL), and indirect bilirubin (IBIL) were determined at 25°C by standard enzymatic techniques (micro method, Kodak Ektachem, Germany).

### Measurement of the weight and volume of liver

After the observation period, liver tissues from the rat model dual grafts were collected and weighed using an electronic balance scale, and volumes were measured by incorporating the use of a measuring cylinder.

### Hematoxylin and eosin staining

Specimens were fixed in 10% buffered formaldehyde phosphate for at least a day for tissue evaluation and were subsequently dehydrated and embedded in paraffin wax to cut sections at a thickness of 4 µm. The growth status of bilateral grafts was evaluated. Morphologic parameters were recorded by routine histology.

### Real-time polymerase chain reaction

RNA was isolated using TRIzol (Invitrogen). A Reverse Transcription Reagent kit with gDNA Eraser (Takara Bio, Shiga, Japan) was used to perform reverse transcription. Real-time polymerase chain reaction (PCR) was performed using an ABI 7500 Sequence Detection System (Applied Biosystems, Foster City, CA) with SYBR Green I (Takara Bio, Shiga, Japan). Data were analyzed using the comparative method (2^−ΔΔCT^). The primers were purchased from the Beijing Genomics Institute (Shenzhen, China), and the primer sequences are shown in **Table 3**.

**Table 3:** Primer sequences of genes.

β-Actin forward primer (rat)	5 ′-ACAACCTTCTTGCAGCTCCTC-3 ′
β-Actin reverse primer (rat)	5 ′-AGGATTCCATACCCAGGAAGG-3 ′
Fas forward primer (rat)	5 ′-TCAGCCTGGTGAACGAAAAGT-3 ′
Fas reverse primer (rat)	5 ′-GTTCGTGTGCAAGGCTCAAG-3 ′
Caspase-8 forward primer (rat)	5 ′-CTGCAAGACAACTCGAGCCT-3 ′
Caspase-8 reverse primer (mouse)	5 ′-ATCCGTTCCGTAGACGATGC-3 ′
Caspase-9 f orward primer (rat)	5 ′-TACTCCACCTTCCCAGGTTTTG-3 ′
Caspase-9 f orward primer (rat)	5 ′-AGCAGTGATGCTGGTGTCTG-3 ′
Granzyme b reverse primer (rat)	5 ′-CTCTTGCTCCTGCTGAGCTT-3 ′
Granzyme b forward primer (rat)	5 ′-CTTGGCCTTACTCTTCAGCTTT-3 ′
Caspase-3 reverse primer (rat)	5 ′-GCTTGGAACGGTACGCTAAGA-3 ′
Caspase-3 forward primer (rat)	5 ′-CCCCTTCATCACCATGGCTT-3 ′

### Western blot

Liver tissues were washed with precooled PBS; 600 µl of mixed working solution (RIPA lysis solution: PMSF = 100:1) was added to each sample, ground thoroughly with liquid nitrogen, and centrifuged at 13,000 rpm/min for 10 min. The supernatant was collected and stored in aliquots at −80°C until analysis by western blot analysis. The protein concentration was measured by a BCA protein assay system. Protein samples were separated by 10%–12% SDS-PAGE and transferred to PVDF membranes. The blots were blocked with 5% skimmed milk in Tris-buffered saline containing 0.1% Tween 20 (TBST) for 1 h at room temperature and then incubated at 4°C overnight with primary antibodies against GAPDH (ImmunoWay, 1:8,000), Fas (Proteintech, 1:1,000), caspase-8 (Proteintech, 1:1,000), caspase-9 (Proteintech, 1:800), granzyme b (Abcam, 1:800), and caspase-3 (Abcam, 1:1500). After being washed in TBST three times, the membranes were incubated with a horseradish peroxidase-conjugated secondary antibody (goat anti-rabbit, ImmunoWay, 1:10,000, goat anti-mouse, ImmunoWay, 1:10,000) for 1 h at room temperature. Finally, the membranes were washed three times for 5 min each with TBST. The specific protein bands were visualized using Super Signal West Pico Chemiluminescent Substrate and imaged using a VersaDoc imaging system (Bio-Rad, USA).

### Statistical analysis

The data are shown as the means ± standard deviations (SD). Group comparisons of normally distributed data were performed with unpaired Student’s t test (two-tailed) or one-way ANOVA. Statistical analysis was performed using SPSS 19.0 (IBM, Armonk, NY, USA), and *P* < 0.05 was considered to indicate statistically significant differences.

## Results

### Establishment and optimization of a dual-graft liver transplantation model in rats

**Figure 2:**
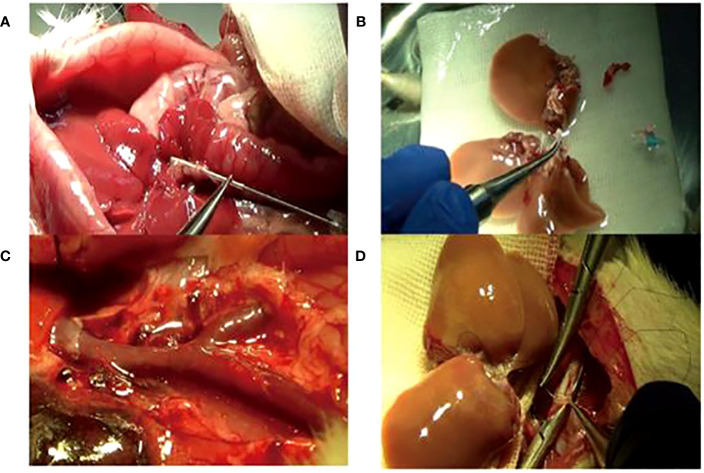
The improved operation techniques. **(A)** Bile duct intubation: the optimum length was 5 mm under the liver. **(B)** Adding the right lateral lobe to the right graft. **(C)** Trimming Y-shaped veins and suturing of small branches of blood vessels. **(D)** Reconstruction of the inferior vena cava by the “triangle pulling method”.

**Figure 3:**
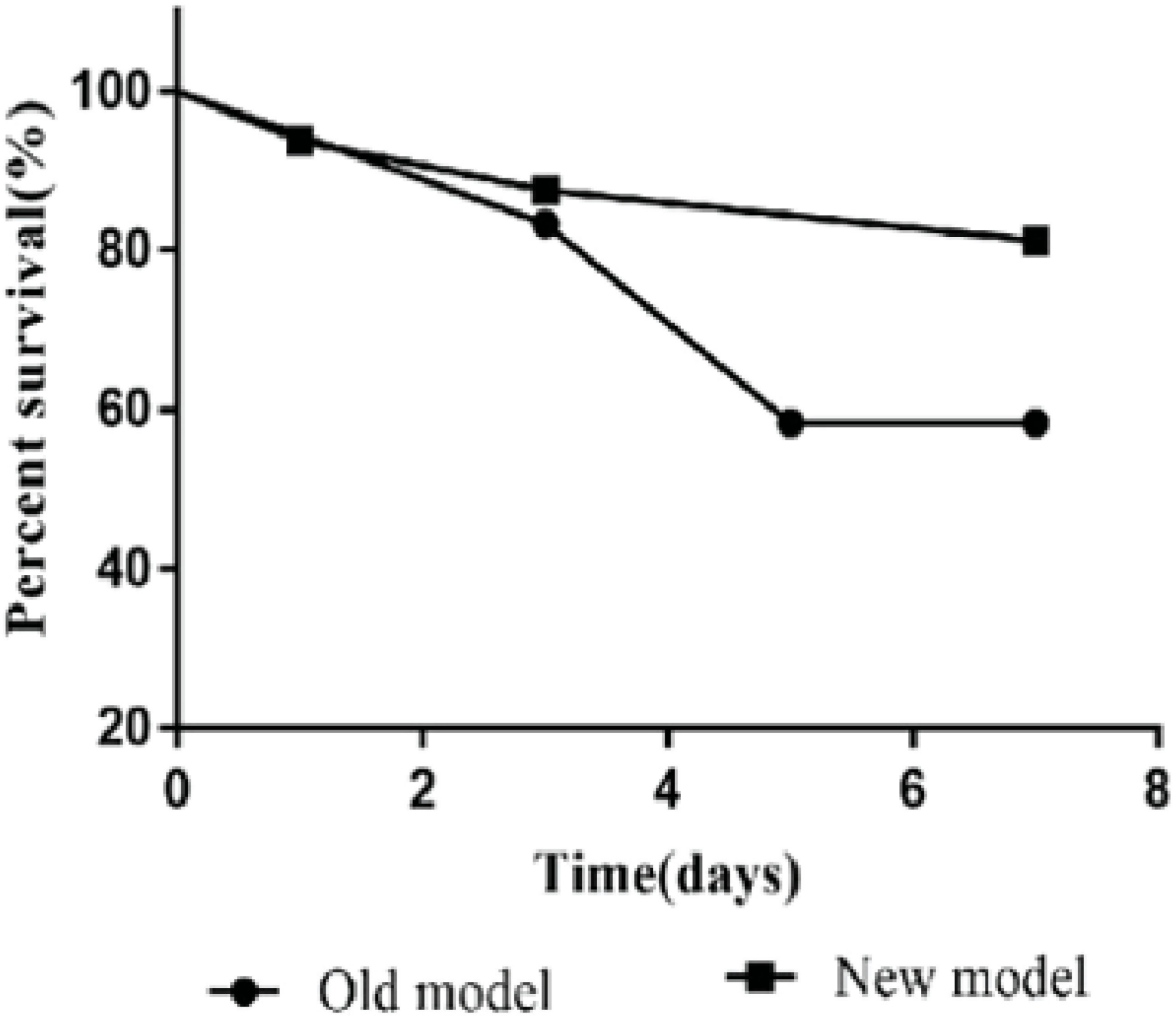
The 7-day survival rate (87.5%) of the new model was higher than that (58.3%) of the old model. Older models have lower survival rates due to a variety of reasons such as biliary leakage, jaundice, and bleeding.

The model’s stability, sustainability, and rigidity were improved by increasing the rat’s body anatomic mass, moderate bile duct length, pruning Y-blood vessels, and “triangle method” anastomosis (**Figure 2**). Compared with a previous model established by our team in 2012 (34), the survival rate of the improved model increased significantly; the 7-day survival rate increased from 58.3% to 87.5%, and the 30-day survival rate was 68.8% (**Figure 3**).

### Volume changes of dual grafts after transplantation

**Figure 4:**
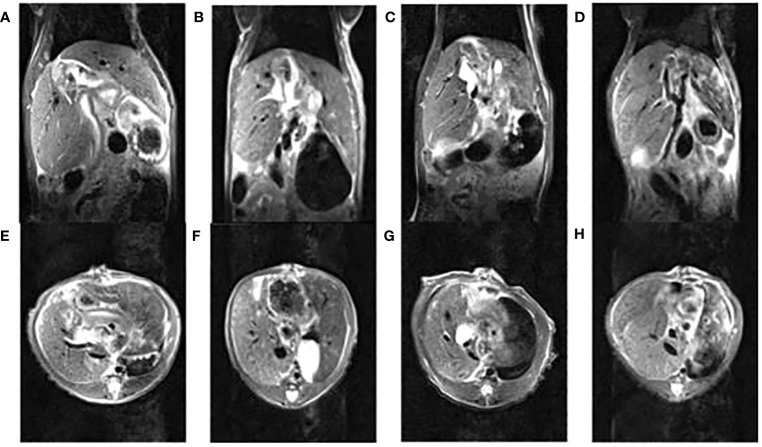
The nuclear magnetic images of dual-graft liver transplantation rats within 30 days, **(A, E)** (1st d), **(B, F)** (3rd d), **(C, G)** (7th d), **(D, H)** (30th d) are coronal and horizontal images, respectively.

The volume of the dual grafts was continuously observed by MRI. The results showed that the volume of the right graft gradually increased, and the left graft firstly increased before the 7th day and then decreased until the 30th day (**Figure 4**).

### Changes in portal vein blood flow in dual grafts

**Figure 5:**
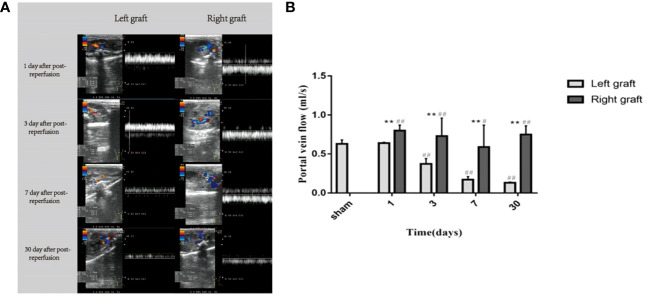
Changes in portal vein flow in dual grafts 1 day, 3 days, 7 days, and 30 days after transplantation. **(A)** Ultrasound images of the dual grafts portal vein. **(B)** Statistical plot of portal vein blood flow (* means statistically significant in right graft vs left graft. *P < 0.05, **P < 0.01. # means statistically significant right and left graft vs control group, #*P* < 0.05, ##*P* < 0.01, n = 6).

The portal vein blood flow of the dual grafts were continuously observed by US. According to the blood flow formula Q = Vmean×π×(D/2)^2^ (Q means quantity of flow, V means velocity, D means diameter), the portal vein blood flow was calculated. The results showed that the PVF of the left graft gradually decreased until the 30th day, while right graft decreased slightly before the 7th day but increased significantly on the 30th day (**Figure 5**).

### Serological changes in rats after dual-graft liver transplantation

**Figure 6:**
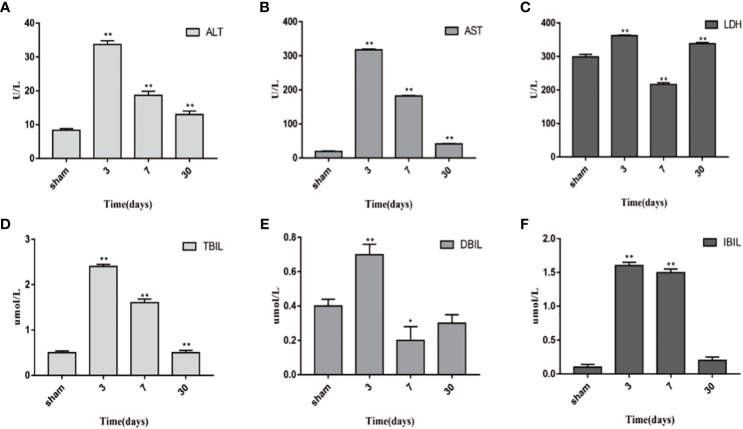
Changes in blood biochemical indicators 1 day, 3 days, 7 days, and 30 days after transplantation. **(A)** ALT. **(B)** AST. **(C)** LDH. **(D)** TBIL. **(E)** DBIL. **(F)** IBIL. *P<0.05, **P<0.01, ***P<0.001.

ALT, AST, TBIL, DBIL, IBIL, and LDH are significant indexes for liver function evaluation. Blood samples from the sham-operated group and transplanted group on the 3th, 7th, and 30th days were obtained. The results are shown in **Figure 6**. Compared with the sham-operated group, liver function increased at different time points in transplanted group, and the differences in liver function indexes were statistically significant (*P* < 0.05). ALT, AST, TBIL, DBIL, IBIL, and LDH all peaked on day 3 and then decreased continuously. Moreover, the liver function parameters recovered, while LDH increased again drastically on the 30th day.

### Morphological measurement of the weight and volume of dual grafts

**Figure 7:**
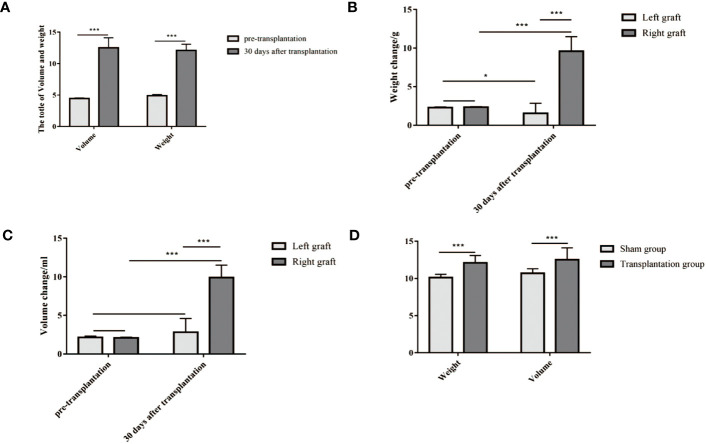
**(A–C)** The weight and volume of grafts both increased 30 days after transplantation compared to their initial weight and volume. **(D)** The level of weight and volume in the transplantation group was similar to that in the sham group. **(A)** The volume and weight of dual grafts (weight, 12.09 ± 1 g vs 4.91 ± 0.18 g; volume, 12.52 ± 1.60 ml vs 4.47 ± 0.08 ml). **(B)** The weight of a single graft (right, 9.61 ± 1.87 g vs 2.34 ± 0.07 g; left, 1.54 ± 1.30 g vs 2.30 ± 0.07 g). **(C)** The volume of a single graft (9.91 ± 1.61 ml vs 2.09 ± 0.08 ml; left, 2.81 ± 1.79 ml vs 2.15 ± 0.17 ml). **(D)** Weight and volume in the transplantation group and sham group (weight, 12.09 ± 1.00 g vs 10.1 ± 0.42 g; volume, 12.53 ± 1.60 ml vs 10.71 ± 0.59 ml). *P<0.05, **P<0.01, ***P<0.001.

The weight and volume of dual grafts were measured before the operation and 30 days after transplantation. Compared with the initial weight and volume of the two grafts, the total weight and total volume after 30 days of transplantation increased significantly (*P* < 0.05) (**Figure 7A**). The weight and volume of the right graft were significantly larger than those of the left graft (**Figures 7B, C**), compared with the sham operation group. There was no significant difference in total volume masses 30 days post transplantation (*P* < 0.05) (**Figure 7D**).

### Hematoxylin and eosin staining

**Figure 8:**
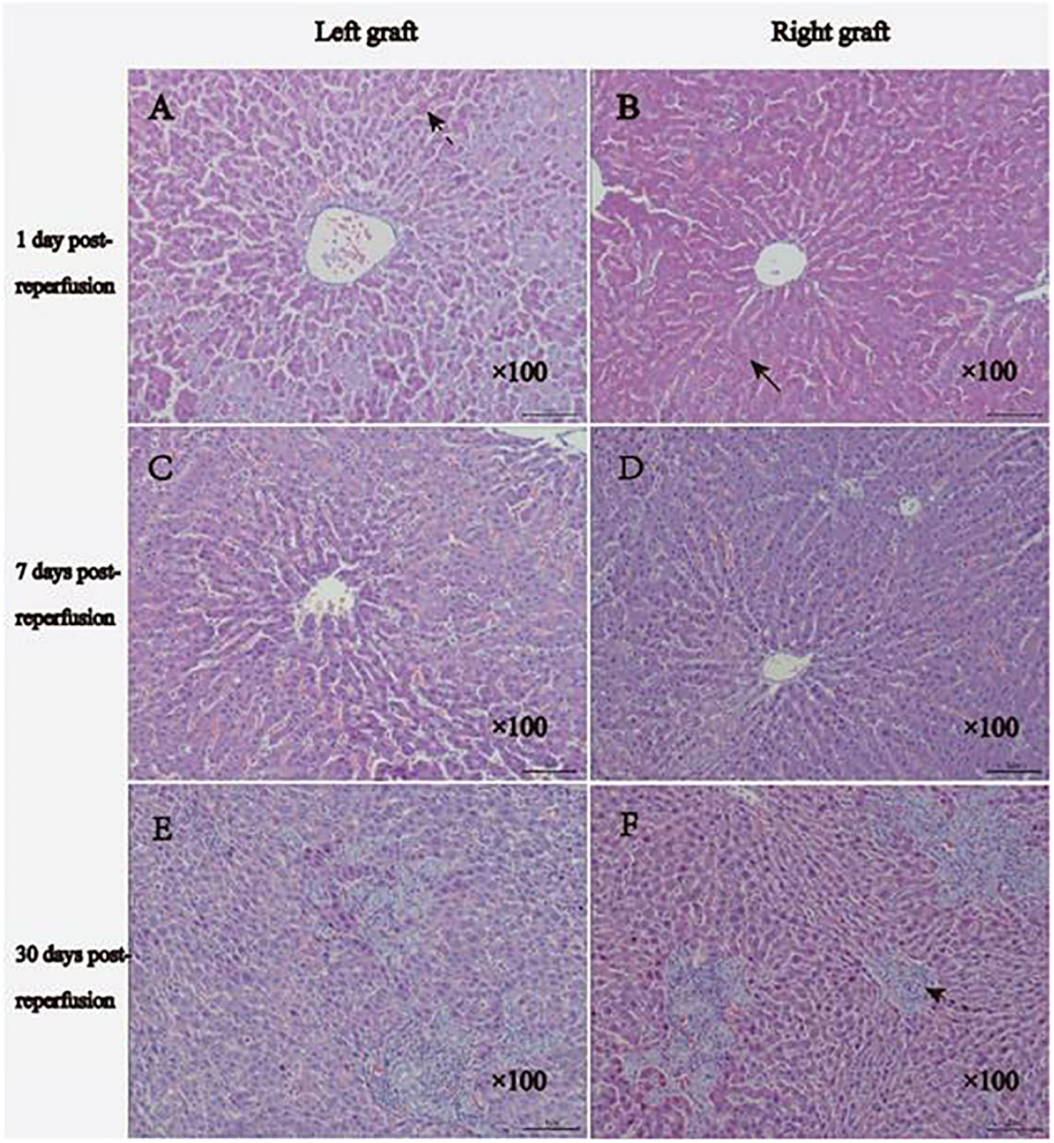
Histology results of HE staining. **(A, B)**, 1 day after reperfusion: the right graft shows moderate vacuolar changes, and the left graft shows moderate-severe vacuolar changes. Two grafts showed sinusoidal congestion and swollen endothelial cells. **(C, D)** 7 days after reperfusion: reduced vacuolar changes and bile duct hyperplasia were observed on both grafts. **(E, F)**, 30 days after reperfusion, the morphology of hepatocytes of the two grafts tended to be normal, significant hyperplasia of the bile duct and fibrous tissue was observed, and more inflammatory cell infiltration could be observed in the portal region of the left graft. The normal arrow indicates vacuolar changes, the broken arrow displays sinusoidal congestion, and the dotted arrow indicates hyperplasia of the bile duct.

In the early stages following transplantation, the dual grafts showed vacuolar changes, sinusoidal congestion, and swelling of the endothelial cells in the portal area. The vacuolated lesions were gradually alleviated. Nonetheless, up to 30 days post-transplantation, the hepatocyte morphology of the dual grafts seemed to be normal, and significant hyperplasia of the bile duct was observed; this suggested considerable growth in the liver tissue. In addition, there were a large number of infiltrating inflammatory cells in the portal area of the left graft (**Figure 8**).

### Changes in the apoptotic indexes in dual grafts

**Figure 9:**
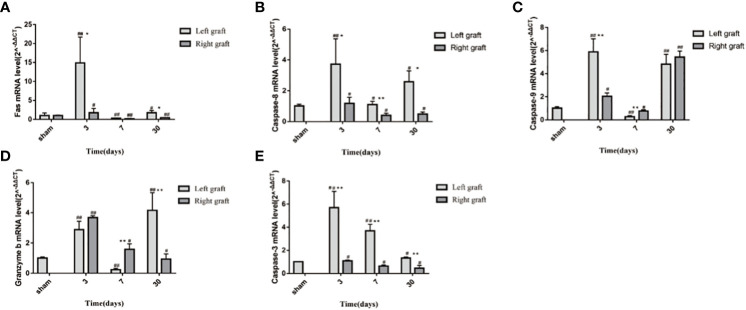
The mRNA changes in apoptosis indexes of dual grafts. **(A)** Fas; **(B)** Caspase-8; **(C)** Caspase-9; **(D)** Granzyme b; **(E)** Caspase-3. * means statistically significant in Right graft VS Left graft. *P<0.05, **P<0.01, ***P<0.001. # means statistically significant Right and Left graft VS control group, #P<0.05, ##P<0.01, ###P<0.001. (n=6).

**Figure 10:**
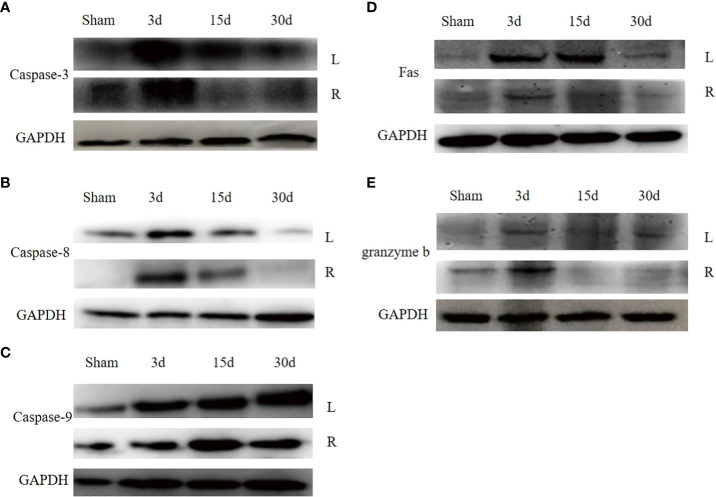
**(A)** The apoptosis protein expression in dual grafts. **(A)**, Caspase-3. **(B)**, Caspase-8. **(C)**, Caspase-9. **(D)**, Fas. **(E)**, Granzyme b.

To explore the molecular mechanisms of dual- graft volume differences after DGLT, we compared the apoptotic changes in dual grafts by qRT-PCR and Western blots in the sham group and transplantation group on the 3rd, 7th, and 30th days post-surgery. RT-PCR showed that fas, caspase-8, granzyme b, and caspase-3 were highly expressed on the 3rd day and then decreased gradually until the 30th day in the right graft, and caspase-9 increased again on the 30th day. In the left graft, except caspase-3 that increased on the 3rd day and decreased until the 30th day, all others increased on the 3rd day, decreased on the 7th day, and increased again on the 30th day. Overall, apoptosis was higher in the left graft than the right graft (**Figure 9**). Western blot analysis showed that fas, caspase-8, and caspase-3 firstly increased and then decreased in dual grafts. However, the expression of granzyme b and caspase-9 continued to increase in the left grafts and decreased in the right graft from the 7th day to the 30th day (**Figure 10**).

## Discussion

The donor shortage puts patients awaiting liver transplantation at risk of death. The emergence of split and living liver transplantation largely expands the donor pool, but SFSS occurs when donor–recipient volume is unmatched or when donor liver quality is poor (35). DGLT surgery has been shown to be effective in solving SFSS (13). However, after DGLT, some patients have shown unilateral graft atrophy and even death (13, 20, 24, 27, 28), which is an undesirable complication for liver transplantation. Moreover, the small number of cases and short follow-up time lead to a lack of information on the physiological process in DGLT. Thus, our team has established a rat dual-graft liver transplantation model (34, 36). We improved the stability and observed the changes in dual- graft post-transplantation to explore the mechanism of volume unbalance.

There are some differences when comparing the DGLT rat model and the clinical DGLT. First and foremost, the rat model has dual donors from the right upper lobe of the rat’s liver and volume difference between the two grafts is not quite vast. However, in a clinical scenario, there are two types: dual left lobe liver transplantation and left lobe plus right lobe transplantation. In dual left lobe transplantation, there comes a big question as to which one of the two left lobes should be placed in the hepatic fossa and which one should be placed in the left hepatic fossa. The number and size of the bile ducts of the graft are the basis; the liver lobe with multiple bile ducts or the liver lobe whose bile duct size does not match the recipient bile duct is usually placed on the left side because multiple bile duct-to-bile duct anastomoses are performed on the right side. If both left lobes are single bile ducts, then the evaluation would be based on the number and size of hepatic arteries. If the conditions of the two left liver lobes are the same, the dominant liver lobe should be placed in situ. On the left, if the volume is larger, the degree of steatosis is lower. Both left lobe and right lobe transplantations were orthotopic transplantation. However, in the rat DGLT model, due to the limitation of microsurgery technology, bile duct–bile duct anastomosis is always completed through a Y-type prosthesis, portal vein anastomosis is completed through the iliac blood vessel, and the hepatic artery is directly ligated. However, clinically, the portal vein and hepatic artery are easy to operate by vascular anastomosis (26, 34).

After succeeding the establishment of a stable model, we used MRI to observe the volume change in dual grafts continuously and dynamically. The growth of right grafts was faster than that of left grafts, and the volume of left grafts increased slowly, which seems to reproduce the occurrence of graft atrophy cases in clinical DGLT. In clinical DGLT, cadaveric liver, left graft, small initial graft, and left liver lobe transplanted to right hepatic fossa were prone to atrophy. (1) Atrophy of the cadaveric liver as a donor may be associated with the long preservation time and impaired liver function (13). (2) The donor’s left hepatic lobe inserted into the recipient’s right hepatic fossa is prone to atrophy, mainly because it is not *in situ* and it causes tension, distortion, and stretching during surgical operation (23). (3) For smaller graft before transplantation, some scholars believe that larger grafts easily gain more access to portal blood flow and grow well (21, 23).

To explore the cause of volume unbalance of dual grafts, we used US to detect PVF of dual grafts in the receptor and found that there were PVF differences in dual grafts. The PVF was consistently decreased in the left graft. In the right graft, although the PVF decreased on the 3th (0.37 ± 0.07 ml/s vs 0.73 ± 0.23 ml/s, left side vs. right side) and 7th (0.17 ± 0.04 ml/s vs 0.59 ± 0.28 ml/s, left side vs. right side) days after surgery, it was still more than the left, and the right graft PVF increased on the 30th day, which may be a contributing factor to volume differences in dual grafts. Broering et al. and Kaihara et al. believed that the growth of the dual grafts is related to the distribution of PVF and that larger grafts have an advantageous growth and preferentially obtain more blood from the ipsilateral portal vein, smaller grafts obtain less PVF, and proliferation is slower. The larger-volume graft seizes blood flow from the small graft, which is called the “blood stealing phenomenon” (21, 23). Some scholars also believe that hepatic vein stenosis is related to graft proliferation and shrinkage, increased hepatic venous pressure, and the ipsilateral portal vein pressure resulted in decreased blood perfusion of the graft and increased portal vein perfusion of the contralateral graft (30, 33).

Apoptotic indicators of the left graft were higher than the right graft. In the right graft, the apoptosis level decreased except caspase-9 that decreased on the 7th day and increased on the 30th day. In the left graft, Fas, caspase-8, caspase-9, and granzyme b decreased on the 7th day and increased on the 30th day, which was consistent with the volume change of the grafts and peaked on the 7th day, potentially explaining that the volume changes may be related to apoptosis. However, we did not compare the left and right grafts by western blotting.

Serological changes of liver function showed the worst liver function on the 3th day after transplantation and then gradually recovered on day 7 and day 30, which was similar to the changes of apoptosis indicators. However, LDH increased again drastically on the 30th day. In fact, after day 7, we also performed two additional time points at day 15 and day 25, but the changes were not significant, and we considered that continuous anesthesia might be the cause of the changes in LDH.

In addition, we measured the mass and volume of the dual graft on the 30th day. Compared with the grafts before transplantation, the total mass and volume of the transplantation group reached the level of the sham operation group and even slightly exceeded the sham operation group. Furthermore, with later observations, we found that many rats have unilateral graft atrophy, and even though the left graft diminished, the receptors were healthy. Therefore, although atrophy was not the expected result, it did not seem to affect the survival of the rats.

Although the animal model was successfully established and optimized in this study, the transplantation mode was all dual right liver lobe, which was different from the complex liver lobe combination pattern of the clinical scene and only initially reproduced the graft atrophy scenario after clinical DGLT. Secondly, the mechanism exploration is also very shallow, and the model observation time is not long enough. The operation is also complicated, and it is difficult to popularize this model for the study of liver transplantation. In addition, although some patients have unilateral graft atrophy after DGLT, the survival rate of most patients is tremendously good and no uncurable complications can be pinpointed (37, 38), similar to the rat model’s survival rate in our experiment. This “extra” or atrophic graft, although it failed to regenerate well eventually, seems to support patients as a transition through the period of greatest need for liver (14, 19). In the future, we will further optimize this animal model to explore the mechanism of cooperative regeneration of the atrophic graft with another graft to achieve patient health.

## Data availability statement

The raw data supporting the conclusions of this article will be made available by the authors, without undue reservation.

## Ethics statement

The animal study was approved by Lanzhou University Second Hospital Institutional Animal Care and Use committee. The study was conducted in accordance with the local legislation and institutional requirements.

## Author contributions

Contributions: (I) Conception and design: HC and YZ; (II) Administrative support: HC; (III) Experiment: DW, YM, and BG; (IV) Provision of study materials: DW and XL; (V) Collection and assembly of data: DW, YY, and YZ; (VI) Data analysis and interpretation: All authors; (VII) Manuscript writing: DW; (VIII). All authors contributed to the article and approved the submitted version.

## References

[1] Von Felden J Villanueva A. Role of molecular biomarkers in liver transplantation for hepatocellular carcinoma. Liver Transpl (2020) 26(6):823–31. doi: 10.1002/lt.25731 32061009

[2] Cornide-Petronio ME Jiménez-Castro MB Gracia-Sancho J Peralta C.. New insights into the liver-visceral adipose axis during hepatic resection and liver transplantation. Cells (2019) 8(9):1100. doi: 10.3390/cells8091100 31540413 PMC6769706

[3] Abreu P Gorgen A Oldani G Hibi T Sapisochin G. Recent advances in liver transplantation for cancer: The future of transplant oncology. JHEP Rep (2019) 1(5):377–91. doi: 10.1016/j.jhepr.2019.07.004 PMC7005652 32039389

[4] Rela M Reddy MS. Living donor liver transplant (LDLT) is the way forward in Asia. Hepatol Int (2017) 11(2):148–51. doi: 10.1007/s12072-016-9780-z 28097531

[5] Lan X Zhang H Li HY Chen KF Liu F Wei YG. Feasibility of using marginal liver grafts in living donor liver transplantation. World J Gastroenterol (2018) 24(23):2441–56. doi: 10.3748/wjg.v24.i23.2441 PMC6010938 29930466

[6] Hughes CB Humar A. Liver transplantation: current and future. Abdom Radiol (NY) (2020) 46(1):2–8. doi: 10.1007/s00261-019-02357-w 31953588

[7] Yoon YI Lee SG. Living donor liver transplantation for hepatocellular carcinoma: an asian perspective. Dig Dis Sci (2019) 64(4):993–1000. doi: 10.1007/s10620-019-05551-4 30895483

[8] Samonakis DN Germani G Burroughs AK. Immunosuppression and HCV recurrence after liver transplantation. J Hepatol (2012) 56(4):973–83. doi: 10.1016/j.jhep.2011.06.031 21963518

[9] Shazi L Abbas Z. Ethical dilemmas related to living donor liver transplantation in Asia. Ir J Med Sci (2019) 188(4):1185–9. doi: 10.1007/s11845-019-01989-7 30798504

[10] Goldaracena N Barbas AS. Living donor liver transplantation. Curr Opin Organ Transplant (2019) 24(2):131–7. doi: 10.1097/MOT.0000000000000610 30694993

[11] Masuda Y Yoshizawa K Ohno Y Mita A Shimizu A Soejima Y. Small-for-size syndrome in liver transplantation: Definition, pathophysiology and management. Hepatobiliary Pancreat Dis Int (2020) 19(4):334–41. doi: 10.1016/j.hbpd.2020.06.015 32646775

[12] Lu H Lu L Zhang F Zhai Y Wang X. Living donor liver transplantation: where do we stand and where are we going? Hepatobiliary Surg Nutr (2016) 5(2):141–4. doi: 10.3978/j.issn.2304-3881.2015.10.02 PMC4824740 27115008

[13] Lee SG Hwang S Park KM Kim KH Ahn CS Lee YJ. Seventeen adult-to-adult living donor liver transplantations using dual grafts. Transplant Proc (2001) 33(7-8):3461–3. doi: 10.1016/S0041-1345(01)02491-5 11750481

[14] Chen H Zhang Y Han YM Huguet E Huang DS Dong JH. Dual liver transplantation. J Zhejiang Univ Sci B (2013) 14(3):178–84. doi: 10.1631/jzus.B1200041 PMC3596568 23463760

[15] Campos BD Botha JF. Strategies to optimize donor safety with smaller grafts for adult-to-adult living donor liver transplantation. Curr Opin Organ Transplant (2012) 17(3):230–4. doi: 10.1097/MOT.0b013e32835365b2 22569511

[16] Ibrahim S Chen CL Wang CC Wang SH Lin CC Liu YW. Small remnant liver volume after right lobe living donor hepatectomy. Surgery (2006) 140(5):749–55. doi: 10.1016/j.surg.2006.02.019 17084717

[17] Dayangac M Taner CB Akin B Uraz S Balci D Duran C. Dual left lobe living donor liver transplantation using donors unacceptable for right lobe donation: a case report. Transplant Proc (2010) 42(10):4560–3. doi: 10.1016/j.transproceed.2010.09.149 21168737

[18] Zhang Y Wen T Chen Z Yan L Li B Zeng Y. Following up of liver transplantation using dual left grafts from living donors–one case. Hepatogastroenterology (2008) 55(81):235–6. 18507114

[19] Xu Y Chen H Yeh H Wang H Leng J Dong J. Living donor liver transplantation using dual grafts: Experience and lessons learned from cases worldwide. Liver Transpl (2015) 21(11):1438–48. doi: 10.1002/lt.24315 26336078

[20] Chen Z Yan L Li B Zeng Y Wen T Zhao J. Prevent small-for-size syndrome using dual grafts in living donor liver transplantation. J Surg Res (2009) 155(2):261–7. doi: 10.1016/j.jss.2009.01.001 19481224

[21] Broering DC Walter J Rogiers X. The first two cases of living donor liver transplantation using dual grafts in Europe. Liver Transpl (2007) 13(1):149–53. doi: 10.1002/lt.21042 17192855

[22] Morita Y Kariya T Nagai S Itani A Isley M Tanaka K. Hepatic vein flow index during orthotopic liver transplantation as a predictive factor for postoperative early allograft dysfunction. J Cardiothorac Vasc Anesth (2021) 35(11):3275–82. doi: 10.1053/j.jvca.2020.12.034 33455886

[23] Kaihara S Ogura Y Kasahara M Oike F You Y Tanaka K. A case of adult-to-adult living donor liver transplantation using right and left lateral lobe grafts from 2 donors. Surgery (2002) 131(6):682–4. doi: 10.1067/msy.2002.123801 12075185

[24] Yang CH Chen CL Wang CC Concejero AM Wang SH Liu YW. Dual grafts in adult-to-adult living donor liver transplantation: a single center experience in Taiwan. Surgery (2009) 145(2):212–8. doi: 10.1016/j.surg.2008.09.008 19167977

[25] Hwang S Lee SG Moon DB Song GW Ahn CS Kim KH. Exchange living donor liver transplantation to overcome ABO incompatibility in adult patients. Liver Transpl (2010) 16(4):482–90. doi: 10.1002/lt.22017 20222052

[26] Song GW Lee SG Moon DB Ahn CS Hwang S Kim KH. Dual-graft adult living donor liver transplantation: an innovative surgical procedure for live liver donor pool expansion. Ann Surg (2017) 266(1):10–8. doi: 10.1097/SLA.0000000000001776 27192349

[27] Lu CH Chen TY Huang TL Tsang LL Ou HY Yu CY. Regeneration and outcome of dual grafts in living donor liver transplantation. Clin Transplant (2012) 26(2):E143–8. doi: 10.1111/j.1399-0012.2012.01621.x 22432787

[28] Lu Q Wu H Yan LN Chen ZY Fan YT Luo Y. Living donor liver transplantation using dual grafts: ultrasonographic evaluation. World J Gastroenterol (2010) 16(31):3979–83. doi: 10.3748/wjg.v16.i31.3979 PMC2923774 20712061

[29] Botea F Braşoveanu V Constantinescu A Ionescu M Matei E Popescu I. Living donor liver transplantation with dual grafts – a case report. Chirurgia (Bucur) (2013) 108(4):547–52. 23958100

[30] Ko EY Kim TK Kim PN Kim AY Ha HK Lee MG. Hepatic vein stenosis after living donor liver transplantation: evaluation with Doppler US. Radiology (2003) 229(3):806–10. doi: 10.1148/radiol.2293020700 14576444

[31] Balci D Kirimker EO. Hepatic vein in living donor liver transplantation. Hepatobiliary Pancreat Dis Int (2020) 19(4):318–23. doi: 10.1016/j.hbpd.2020.07.002 32709407

[32] Nicoluzzi JE Silveira F Silveira FP Macri MM Monteiro M Woitovicz V. [The first dual left lobe adult-to-adult liver transplantation in Brazil]. Rev Col Bras Cir (2012) 39(3):226–9. doi: 10.1590/S0100-69912012000300012 22836573

[33] Scheinfeld MH Bilali A Koenigsberg M. Understanding the spectral Doppler waveform of the hepatic veins in health and disease. Radiographics (2009) 29(7):2081–98. doi: 10.1148/rg.297095715 19926763

[34] Zhang Y He Y Praseedom RK. Establishment of animal model of dual liver transplantation in rat. PloS One (2012) 7(7):e40818. doi: 10.1371/journal.pone.0040818 22829887 PMC3400666

[35] Terrault NA Francoz C Berenguer M. Liver transplantation 2023: status report, current and future challenges. Clin Gastroenterol Hepatol (2023) 21(8):2150–66. doi: 10.1016/j.cgh.2023.04.005 37084928

[36] Xie XJ Ye YF He Y. Development of an improved rat model of dual graft liver transplantation with long-term survival. Genet Mol Res (2014) 13(3):8035–45. doi: 10.4238/2014.September.29.16 25299118

[37] Vinayak N Ravi M Ankush G. Dual graft living donor liver transplantation - a case report. BMC Surg (2019) 19(1):149. doi: 10.1186/s12893-019-0606-5 31640624 PMC6805583

[38] Petrowsky H. Avoiding dual graft loss in simultaneous liver retransplantation and primary kidney transplantation. Transplantation (2020) 104(7):1328–9. doi: 10.1097/TP.0000000000003036 31651795

